# Assessing Intraspecific Variation in Effective Dispersal Along an Altitudinal Gradient: A Test in Two Mediterranean High-Mountain Plants

**DOI:** 10.1371/journal.pone.0087189

**Published:** 2014-01-29

**Authors:** Carlos Lara-Romero, Juan J. Robledo-Arnuncio, Alfredo García-Fernández, Jose M. Iriondo

**Affiliations:** 1 Departamento de Biología y Geología, Universidad Rey Juan Carlos, Madrid, Spain; 2 Department for Ecology and Forest Genetics, INIA-CIFOR, Madrid, Spain; 3 Institut Botanic de Barcelona, IBB-CSIC-IQUB, Barcelona, Spain; CNR, Italy

## Abstract

**Background:**

Plant recruitment depends among other factors on environmental conditions and their variation at different spatial scales. Characterizing dispersal in contrasting environments may thus be necessary to understand natural intraspecific variation in the processes underlying recruitment. *Silene ciliata* and *Armeria caespitosa* are two representative species of cryophilic pastures above the tree line in Mediterranean high mountains. No explicit estimations of dispersal kernels have been made so far for these or other high-mountain plants. Such data could help to predict their dispersal and recruitment patterns in a context of changing environments under ongoing global warming.

**Methods:**

We used an inverse modelling approach to analyse effective seed dispersal patterns in five populations of both *Silene ciliata* and *Armeria caespitosa* along an altitudinal gradient in Sierra de Guadarrama (Madrid, Spain). We considered four commonly employed two-dimensional seedling dispersal kernels exponential-power, 2Dt, WALD and log-normal.

**Key Results:**

No single kernel function provided the best fit across all populations, although estimated mean dispersal distances were short (<1 m) in all cases. *S. ciliata* did not exhibit significant among-population variation in mean dispersal distance, whereas significant differences in mean dispersal distance were found in *A. caespitosa*. Both *S. ciliata* and *A. caespitosa* exhibited among-population variation in the fecundity parameter and lacked significant variation in kernel shape.

**Conclusions:**

This study illustrates the complexity of intraspecific variation in the processes underlying recruitment, showing that effective dispersal kernels can remain relatively invariant across populations within particular species, even if there are strong variations in demographic structure and/or physical environment among populations, while the invariant dispersal assumption may not hold for other species in the same environment. Our results call for a case-by-case analysis in a wider range of plant taxa and environments to assess the prevalence and magnitude of intraspecific dispersal variation.

## Introduction

Dispersal has important implications for plant species. It determines gene flow rates and the spatial distribution of genetic diversity [1]–[3], and it influences processes like colonization [4], range expansion [5], local adaptation [6] and recruitment patterns [7]. It also affects metapopulation dynamics and species coexistence and diversity [8], [9].

Dispersal studies distinguish between primary and effective dispersal. Primary dispersal is the initial movement of seeds to the location where they are first deposited, whereas effective dispersal is the complex result of primary dispersal and the post-dispersal processes that take place after the seed is deposited on the soil surface until it is a successfully established seedling [7], [10]. Effective dispersal is more relevant to population dynamics, because it comprises the environmental factors that are needed for seedling recruitment [7], [10], [11].

The fecundity of reproductive adults and the distribution of dispersal distances are fundamental characteristics of the dispersal process which have been extensively used as basic descriptors of this process [7], [12]–[14]. However, in many systems, measuring seed production and dispersal distances poses a challenge, because dispersing seeds cannot be tracked easily, and seed or seedling shadows of neighboring plants typically overlap, making it difficult to identify mother plants [15]. Inverse modelling is a useful methodology that allows the fecundity of adult plants and the shape of the dispersal kernel (the probability density function of propagule dispersal distances from an individual plant) to be estimated without identifying the exact source of each seed or seedling [12], [13], [16]–[18]; reviewed in [19].

Most seed dispersal research, employing inverse modelling or other methods, has been carried out in single populations, disregarding potentially relevant environmental variation across space that could affect the natural processes underlying the recruitment of particular species [10], [19]; but see [5], [20]. Heterogeneity in environmental conditions and landscape properties (e.g. intra and interspecific plant density, fragmentation, soil moisture, wind conditions) may indeed influence the dispersal kernel even over local geographic scales [10], [17], [19], [21]. However, this variation is implicitly ignored in spatially unreplicated studies, which may lead to local results that are unrepresentative of the average dispersal pattern of the species. Hence, more comparative empirical studies are needed to test whether effective dispersal kernels exhibit intraspecific variation across contrasting environments and to find environmental correlates of potential variation [10], [19], [22]. Such studies could assess the validity of kernel-based approaches that assume a sole kernel for each species [22].

High-mountain habitats have been identified as one of the most fragile environments in the world, and global warming is thought to be especially critical for plant populations in mountain systems [23], [24]. The movement of plants to higher elevations tracking their climatic niche seems to be one of the main responses to ongoing global warming [25], [26], although phenotypic plasticity and adaptation may also play an important role in their response [27]–[29]. Although all these processes are significantly conditioned by dispersal and recruitment patterns [4]–[6], [10], [27], no explicit measurements of dispersal kernels have been made for high-mountain plants so far. A better understanding of dispersal and recruitment patterns of high-mountain plants through the study of effective dispersal kernels would, therefore, provide greater insight into high-mountain plant species response to climate warming.

We used an inverse modelling approach to measure the net reproductive rate and effective dispersal kernel parameters along an altitudinal gradient in a threatened Mediterranean high-mountain pasture community in central Spain. Analyses were performed on five populations of each of two representative species of the community: *Silene ciliata* Poiret and *Armeria caespitosa* (Gómez Ortega) Boiss. in DC. Previous studies carried out on these species showed that the altitudinal gradient is associated with an environmental stress gradient, with the lowest population experiencing the most stressful conditions, constraining seedling establishment and reproductive performance [30]–[34]. We expected that variation in population structure and physical environment along the altitudinal gradient could modify the spatial recruitment pattern and dispersal distance of the species. Specifically, we addressed two main questions: (1) What is the spatial range of effective seed dispersal in these two high-mountain species? (2) Do effective seed dispersal and fecundity parameters vary among populations at different altitudes?

## Methods

### Ethics Statement

All necessary permits were obtained for the described field studies. Mr. Juan Antonio Vielva from the Administration Bureau of the Natural Park of Cumbres, Circo y Lagunas de Peñalara and Mr. Antonio Sanz from the Administration Bureau of the Regional Park of Cuenca Alta del Manzanares gave their permission to work in the protected natural areas. Field studies did not involve any endangered or protected species.

### Study Site and Species

The study was carried out in the orophyllous cryophilic pastures of Sierra de Guadarrama, a mountain range located in central Spain. Mean annual precipitation, measured at the Navacerrada Pass (40° 46′N, 4° 19′W; 1860 m a.s.l.), is 1330 mm with a pronounced dry season (<10% of total annual rainfall) from May to October. Mean annual temperature is 6.3°C, with mean monthly temperatures ranging from −1°C in January to 16°C in July (www.aemet.es). Dry cryophilic pastures occur in the higher summits above the tree line between 1900 and 2430 m.a.s.l and are dominated by *Festuca curvifolia* Lag. ex Lange and other perennial plants interspersed in a shrub matrix characterized by *Cytisus oromediterraneus* Rivas Mart. *et al*. and *Juniperus communis* subsp. *alpina* (Suter) Čelak.

*Silene ciliata* Poiret (Caryophyllaceae) is a chamaephytic cushion perennial plant that occurs in the Mediterranean mountain ranges of southern Europe [35]. One of its southernmost distribution limits is found in Sierra de Guadarrama at altitudes from 1900 to 2430 m, where it grows in dry cryophilic pastures dominated by *Festuca curvifolia*. It blooms in late summer, with a peak in early August [36]. Flowering stems reach 15 cm in height and have 1–5 flowers. Fruit capsules have up to 100 seeds which are wind dispersed in August-September. *Silene ciliata* seeds have an average mass of 0.59 mg and their diameter ranges between 1.1 and 1.5 mm [37]. The species is essentially barochorous (seeds lack any specific structure to promote dispersal) and is pollinated by syrphid flies, bumblebees and moth species [38]. It is self-compatible, although autogamy is restricted by pronounced protandry [39]. In Sierra de Guadarrama, genetic diversity is quite homogeneous across *S. ciliata* populations, which show significant levels of inbreeding [39].

*Armeria caespitosa* (Gómez Ortega) Boiss. in DC. is a high-mountain dwarf chamaephytic cushion plant, endemic to the mountains of central Spain (Sierra de Guadarrama, Ayllón and East Gredos), which occurs at altitudes from 1600 to 2430 m. It grows in the same dry cryophilic pastures dominated by *Festuca curvifolia*, although isolated individuals have also been found on the ledges of granite and gneiss rocks. Its flowers are grouped in short-scaped flowerheads with 19±8 flowers each. Each flower has a single ovule, which yields one seed that remains enclosed in the papyraceous calyx. Average seed mass is 1.18 mg and its diameter ranges between 2.0 and 4.7 mm [30]. The papyraceous calyx may facilitate seed dispersal by wind [30]. *A. caespitosa* is self-incompatible [31] and pollinated by bees, bumblebees and syrphid flies [31]. Previous studies on the population genetics of the species revealed relatively low genetic differentiation and a complex genetic structure among populations [40], [41].

### Field Data

In August and September 2010, we established 10×10 m plots in five populations of both *S. ciliata* and *A. caespitosa* distributed along an altitudinal gradient at the study site (Table 1). For each plot, we estimated a set of climate variables for the growing season (April-September). We calculated mean, minimum and maximum rainfall and temperature for each population using the Digital Climatic Atlas of Spain of the Spanish Ministry of Environment [42] (Table 1), and mean wind speed at 80 m height using the Spanish Wind Energy Atlas of the Spanish Ministry of Industry (http://atlaseolico.idae.es, Table 1).

**Table 1:** Description of *A. caespitosa* and *S. ciliata* sampling sites.

Population	UTM Coordinates (x,y) m			Altitude m	*Pm* (min, max) mm	*Tm* (min, max) °C	*Ws* m/s	Seedling	Reproductive
*Silene ciliata*									
Peñalara	419427.30	4522814.15		2405	82.50 (32, 149)	8.77 (0.2, 14.1)	10.62	469	1068
Cabezas de Hierro	421169.50	4516859.61		2305	85.17 (34, 151)	8.37 (0.2, 13.7)	10.87	243	1085
Nevero	428863.74	4537167.57		2190	71.00 (30, 121)	9.67 (2.1, 14.6)	8.97	19	245
Najarra	430196.59	4518886.29		2080	72.17 (27, 112)	10.45 (2.8, 15.7)	9	89	523
Laguna	419931.16	4521082.09		1946	77.00 (31, 134)	10.27 (2.8, 15.2)	7.17	6	147
*Armeria caespitosa*									
Cabezas de Hierro	420950.54		4516685.32	2336	85.2 (34, 151)	8.37 (0.2, 13.7)	10.87	145	318
Najarra	430196.79		4518886.33	2080	72.2 (27, 112)	10.45 (2.8, 15.7)	9	498	356
Loma de Cabezas	420013.12		4514794.49	1970	77.0 (30, 131)	9.78 (2.5, 14.8)	8.45	53	132
Collado de las Vacas	419168.95		4513371.38	1882	66.3 (25, 110)	11.80 (4.0, 17.2)	8.46	49	169
Sierra de los Porrones	420580.85		4512251.64	1647	64.5 (26, 102)	12.88 (4.4, 18.3)	7.4	32	52

Geographical coordinates, altitude, climatic variables for the growing season (April-September) and seedling and reproductive plant density (individuals per 10×10 m plot) in study populations. *Pm:* monthly precipitation, *Tm:* monthly temperature, *Ws:* wind speed at 80 m height. Minimum and maximum monthly precipitation and temperatures are provided in brackets.

We mapped every *S. ciliata* and *A. caespitosa* reproductive plant and seedling in each study plot using two high-resolution Differential Global Navigation Satellite System (DGNSS) receivers (Viva GS15, Leica, Switzerland) with an accuracy of 5 cm for *x* and *y* coordinates. We also measured plant diameter and the number of inflorescences of each reproductive plant. To account for potential seedling sources outside the study area, we also recorded the location, diameter and number of inflorescences of reproductive plants in a buffer zone of two meters around the plots. The seedling stage in *S. ciliata* and *A. caespitosa* includes up to one-year individuals (i.e. plants that had germinated in fall or spring and had survived their first full summer), because the seedlings that survive a second growth period grow into the reproductive stage [43], [44]. Summer is critical to the survival of Mediterranean plants due to the incidence of severe droughts [45], [46]. This is also the case in Mediterranean high-mountain plants [47] such as *S. ciliata* and *A. caespitosa* [30], [35]. Within this framework, we included all critical stages of effective dispersal: seed dispersal, seed germination and seedling survival.

The dataset used for this study is available from the institutional repository of Rey Juan Carlos University (BURJC-Digital, http://eciencia.urjc.es/handle/10115/11835).

### Statistical Analysis

We used inverse modelling to estimate seedling dispersal kernel parameters for each population of each species and to test for dispersal parameter variation among populations within species. Following Ribbens *et al.* [13], the expected number of seedling recruits *ŝ_j_* at a given plot or trap *j* of area *A_j_* (*ŝ_j_*) equals the sum of the seedling shadows across this plot of all *m* maternal plants, in the form:
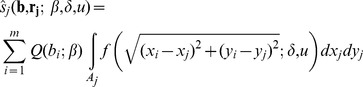
(1a)where **b** and **r_j_** are, respectively, *m*-length vectors of individual plant size measurements *b_i_* and spatial distances *r_ij_* between the spatial coordinates (*x_j_*, *y_j_*) of plant *i*, and the coordinates of plot *j* (*x_i_*, *y_i_*), *Q* is an allometric function with parameter *β* yielding the number of seedlings produced by a single plant, and *f* is a two-dimensional isotropic seedling dispersal probability density function (dispersal kernel) as a function of dispersal distance 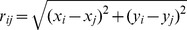 with mean *δ* and shape parameter *u*. The integral in *eqn.* 1*a* provides the exact probability of dispersal from plant *i* into the whole surface *A_j_* of plot *j*. All the studies we are aware of have assumed that the probability of dispersal from any given plant to any of the points lying within area *A_j_* of each plot is constant, yielding the usual simplified expression:
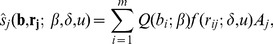
(1b)where *r_ij_* is the spatial distance between plant *i* and the centre of plot *j*. The approximation in *eqn* 1*b* will be closer to the exact values predicted by *eqn*. 1*a* the smaller the seedling plot areas and the longer the distance between maternal plants and seedling plots.

We assumed a linear relationship between seedling production and plant diameter, *Q_i_* = *βb_i_*, because previous studies have shown that plant size is linearly related to seed production in both species [31], [33]. Furthermore, as found in previous studies [14], [48], alternative allometric functions worsened inverse modelling fits, including those using the number of inflorescences (results not shown).

For each population of each species, we divided the 100-m^2^ study area into *c = *100*L* ^−2^ equal adjacent cells of area *L* ^2^, and for each cell *j,* we computed observed (*s_j_*) and predicted (*ŝ_j_*) seedling densities. We used equations 1*a* and 1*b* to estimate *ŝ_j_*, as cell areas were relatively large compared to some of the mother-cell pairwise distances (occasionally with maternal plants lying within the target cell), and it was not obvious *a priori* that the approximation in equation 1*b* would be sufficiently accurate. However, virtually identical dispersal and fecundity estimates were obtained for all populations and assumed kernels using each equation (results not shown). Thus, we only present the results for the latter, whose computational efficiency enabled us to calculate confidence intervals and conduct hypothesis tests (see below) in a reasonable number of CPU hours. We also tested the effect of three different values for cell side length, *L* = 0.125, 0.25 and 0.50 m, on parameter estimates, with lower and upper values approximating DGNSS accuracy and dispersal range, respectively. Distribution of seedling densities across cells exhibited overdispersion relative to a Poisson distribution (*p*<0.05) in all populations except the Nevero population of *S. ciliata*, and we thus modelled seedling counts using a negative binomial distribution [5]:
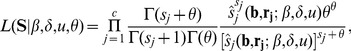
(2)where **S** is the set of *c* observed seedling counts *s_j_*, Γ is the gamma function, and *θ* is the negative binomial parameter, determining the distribution variance (values of *θ* <1 indicate overdispersed distributions, while *θ* >>1 tend to the Poisson distribution). Maximum-likelihood estimates for *β*, *δ*, *u* and *θ* were obtained for each population of each species by maximizing *eqn* 2. Confidence intervals were computed using the profile-likelihood method [49].

As most studies show that no single dispersal function provides consistently superior fits across species [14], [18], [19], [50], the performance of alternative dispersal kernels should always be compared [14], [17]. We considered four commonly employed two-dimensional seedling dispersal kernels, with scale parameter *a* and shape parameter *u*: the 2Dt kernel [12]; see [51] for the parameterization used here.
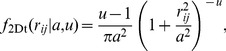
(3)the log-normal kernel [52]

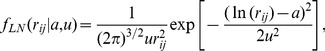
(4)the WALD kernel [14], [53]

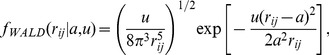
(5)and the exponential-power kernel [5]



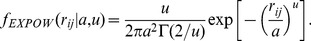
(6)Mean seedling dispersal distances for each kernel are given by *δ*_2Dt_ = *a*Γ(3/2) Γ(*u*−3/2)/Γ(*u*−1), *δ_LN_* = exp (*a* + 0.5*u* ^2^), *δ*_WALD_ = *a* and *δ*_EXPOW_ = *a*Γ(3/*u*)/Γ(2/*u*), respectively. Given that *δ* provides a more intuitive description of dispersal range than *a*, independent of the assumed kernel, we parameterized the model (*eqns* 1 and 2) in terms of *δ* and *u,* rather than in terms of *a* and *u*, facilitating the estimation of profile-likelihood confidence intervals for *δ* [5], [54] and tests of its variation across populations (see below). We used the Akaike Information criterion (AIC) [55] to assess method performance and calculated the goodness of fit of the most parsimonious model by measuring Pearson’s product-moment correlation between observed vs. predicted seedling densities.

We tested for potential differences in dispersal and fecundity parameters across populations using likelihood ratio statistics as in Clark *et al.* [5]. Assuming that mean dispersal distance *δ* is constant across populations within species, we obtained parameter estimates based on the likelihood of the whole species data set **S_q_**, incorporating information from all *q* populations of the species:
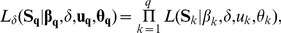
(7)where **β_q_**, **u_q_**, and **θ_q_** are *q*-length vectors of population-specific fecundity parameters *β_k_*, kernel shape parameters *u_k_* and negative binomial parameters *θ_k_*. This model has 3*q* degrees of freedom and provides an average species-level estimate of *δ*. Note that, unlike Clark *et al.* [5], we allowed for different values of the negative binomial parameter (i.e. different degrees of spatial seedling clumping) across populations. We assumed the same kernel family for all populations in conducting this test, choosing the one yielding the best average fit over all populations (i.e. the one with the smallest average AIC across the population-level fits obtained using *eqn*. 2). We then obtained parameter estimates assuming that all parameters (including *δ*) vary among populations within species (still assuming a single consensual kernel family), based on the likelihood
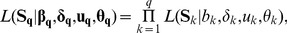
(8)where δq is the q-length vector of population-specific mean dispersal distances δk. The model in *eqn* 8 has 4q−1 degrees of freedom. The deviance
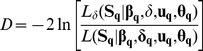
(9)is asymptotically distributed as χ2 with q−1 degrees of freedom. Large deviances mean that stand-specific δ improves the likelihood of the data so substantially that the null hypothesis of invariant δ across populations can be rejected [5]. We first tested for significant differences in δ across all populations of each species. When the overall test was positive, we tested for specific populations with δ-values significantly different from the global species-average by subsequently conducting q deviance tests, one for each population i (χ2 with 1 degree of freedom), using:
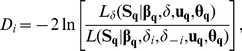
(10)where the numerator is the same likelihood as before, assuming that the mean dispersal distance δ is constant across all populations, while the likelihood in the denominator assumes that the i-th population has a mean dispersal distance δ*_i_*, while the remaining q−1 populations have a constant mean dispersal distance δ_−*i*_. We applied the sequential Bonferroni correction to correct for multiple testing [56]. Analogous tests were conducted to investigate among-population variation in the shape parameter of the dispersal kernel (assuming constant u and variable δ in *eqn* 7) and in the fecundity parameter β (assuming constant β and variable δ in *eqn* 7). Although the field survey at the study sites was exhaustive, some sample sizes were unbalanced and relatively small due to natural density variation (Table 1), which may compromise the large numbers approximation under which the χ2 distribution is expected. Small samples can indeed make likelihood ratio (LR) tests relatively lax in the case of unequal male reproductive success analysis [57]. Smouse et al. [58], proposed the use of nonparametric (permutational) tests as an alternative to LR tests, showing that the former can perform better than LR tests when parent-offspring genealogical information is available. Similar non-parametric tests have not been developed so far in the different statistical framework of inverse modeling, for which LR tests, such as those used here, are still widely employed as feasible approximations for exploratory assessment of variation in dispersal and fecundity parameters [5], [59]–[61]. All statistical analyses were performed in the open source software package R [62].

## Results

We mapped a total of 4095 reproductive individuals and 1603 seedlings across all populations of the two species (Table 1; Figure S1). Reproductive plant and seedling densities varied between populations in both species (Table 1). We focused on the results obtained with a 0.125 m cell side length (Tables 2 and 3), because some of the *S. ciliata* populations did not converge when 0.25 or 0.5 m cell side lengths were used (Table S1). In any case, similar results were obtained for both species for all cell side lengths (Tables 2, 3, S1 and S2). As none of the four models converged for *A. caespitosa* in the Najarra population, this population was excluded from further analyses.

**Table 2:** Estimated parameters for the models fitted to the seedling recruitment data of *S. ciliata*.

Population	Kernel	Mean dispersal(*δ*) (m)		Shape parameter (*u*)		Fecundity parameter(*β*) (seedlings/cm)		Negative binomial parameter *(θ)*		− log*L*	ΔAIC	*r* ^2^
Peñalara	**Log-norm**	**0.32**	**(0.27–0.39)**	**0.64**	**(0.52–0.77)**	**0.009**	**(0.008–0.010)**	**0.359**	**(0.260–0.523)**	**1624.80**	–	**0.12**
	WALD	0.33	(0.28–0.41)	0.64	(0.44–0.99)	0.009	(0.008–0.010)	0.360	(0.260–0.525)	1624.82	0.04	
	2Dt	0.32	(0.27–0.46)	3.80	(1.99–*na*)	0.009	(0.009–0.011)	0.351	(0.255–0.509)	1627.77	5.94	
	Exp-pow	0.31	(0.27–0.37)	1.51	(0.97–2.34)	0.009	(0.008–0.010)	0.349	(0.253–0.507)	1628.76	7.92	
Cabezas de Hierro	**Exp-pow**	**0.37**	**(0.32–0.45)**	**9.86**	**(2.34–** ***na*** **)**	**0.003**	**(0.003–0.004)**	**0.449**	**(0.235–1.198)**	**1029.04**	**–**	**0.05**
	Log-norm	0.43	(0.35–0.60)	0.43	(0.23–0.70)	0.003	(0.003–0.004)	0.460	(0.240–1.246)	1029.04	0.01	
	WALD	0.43	(0.35–0.62)	2.26	(0.84–8.70)	0.003	(0.003–0.004)	0.459	(0.239–1.244)	1029.17	0.27	
	2Dt	0.43	(0.33–0.58)	172.54	(3.65–*na*)	0.003	(0.003–0.004)	0.450	(0.235–1.205)	1031.17	4.25	
Nevero	**Exp-pow**	**0.23**	**(0.17–0.50)**	**2.84**	**(0.74–21.91)**	**0.001**	**(0.001–0.002)**	**142.062**	**(0.045–** ***na*** **)**	**108.04**	–	**0.08**
	2Dt	0.23	(0.17–0.42)	171.06	(1.74–*na*)	0.001	(0.001–0.002)	142.162	(0.082–*na*)	108.12	0.17	
	WALD	0.30	(0.22–0.60)	1.23	(0.41–3.27)	0.001	(0.001–0.002)	141.865	(0.054–*na*)	109.04	1.99	
	Log-norm	0.30	(0.21–0.86)	0.47	(0.29–1.35)	0.001	(0.001–0.002)	50.393	(0.053–*na*)	109.14	2.19	
Najarra	**WALD**	**0.71**	**(0.37–** ***na*** **)**	**0.55**	**(0.27–1.24)**	**0.002**	**(0.003–0.003)**	**0.280**	**(0.092–8.456)**	**461.85**	**–**	**0.04**
	Log-norm	0.62	(0.36–3.01)	0.86	(0.58–1.46)	0.002	(0.002–0.003)	0.280	(0.092–8.401)	462.14	0.59	
	Exp-pow	0.61	(0.36–2.45)	0.83	(0.27–2.49)	0.002	(0.002–0.003)	0.275	(0.091–7.395)	463.15	2.59	
	2Dt	*nc*		*nc*		*nc*		*nc*		*nc*		
Laguna	**Exp-pow**	**0.29**	**(0.17–0.40)**	**1411.75**	**(0.03-** ***na*** **)**	**0.001**	**(0.0003–0.004)**	**0.003**	**(0.001–0.035)**	**38.74**	**–**	**0.01**
	WALD	0.24	(0.14-*na*)	1.00	(*na-na*)	0.001	(0.0003–0.029)	0.004	(0.001–0.049)	38.91	0.34	
	Log-norm	0.24	(0.16-*na*)	0.47	(0.23–*na*)	0.001	(0.0003-*na*)	0.004	(0.001–0.048)	38.95	0.42	
	2Dt	0.23	(0.15–0.86)	165.18	(1.00–*na*)	0.001	(0.0003 - 0.006)	0.003	(0.001–0.035)	39.12	0.76	

For each parameter, 95% confidence intervals are presented along with the mean value. Log-norm, log-normal; Exp-pow, exponential-power. *na* denotes that the confidence interval limit is not available because of flat likelihood function. *nc* denotes models that did not converge.–log*L*, log-likelihood; AIC, Akaike’s information criterion; *r*, Pearson correlation coefficient between observed and predicted seedling density values. Models with the lowest AIC are marked in bold for each population.

**Table 3:** Estimated parameters for the models fitted to the seedling recruitment data of *A. caespitosa*.

Population	Kernel	Mean dispersal(*δ*) (m)		Shape parameter (*u*)		Fecundity parameter(*β*) (seedlings/cm)		Negative binomial parameter *(θ)*		− log*L*	ΔAIC	*r* ^2^
Cabezas de Hierro	**Log-norm**	**0.33**	**(0.24–0.56)**	**0.97**	**(0.78–1.29)**	**0.024**	**(0.019–0.029)**	**0.122**	**(0.076–0.208)**	**584.41**	**–**	**0.17**
	2Dt	0.80	(0.30 - *na*)	1.60	(1.24–2.48)	0.024	(0.020–0.030)	0.122	(0.076–0.207)	584.78	0.73	
	WALD	0.35	(0.24–1.44)	0.21	(0.13–0.35)	0.024	(0.020–0.030)	0.121	(0.076–0.206)	584.80	0.79	
	Exp-pow	0.29	(0.23–0.45)	0.60	(0.31–1.03)	0.024	(0.020–0.029)	0.120	(0.075–0.202)	585.01	1.19	
Loma de Cabezas	**Exp-pow**	**0.66**	**(0.46–1.39)**	**0.69**	**(0.24–2.37)**	**0.014**	**(0.010–0.019)**	**0.061**	**(0.028–0.162)**	**247.54**	**–**	**0.12**
	Log-norm	0.78	(0.48–2.57)	1.02	(0.69–1.64)	0.014	(0.010–0.019)	0.063	(0.028–0.169)	247.82	0.57	
	WALD	0.77	(0.46 - *na*)	0.44	(0.19–1.01)	0.014	(0.010–0.020)	0.064	(0.028–0.171)	247.96	0.85	
	2Dt	0.73	(0.05 - *na*)	2.15	(0.12 - *na*)	0.014	(0.010–0.020)	0.058	(0.026–0.158)	248.64	2.20	
Collado delas Vacas	**2Dt**	**0.30**	**(0.18–** ***na*** **)**	**1.84**	**(1.36–2.85)**	**0.011**	**(0.008–0.014)**	**0.286**	**(0.107–1.330)**	**206.84**	**–**	**0.22**
	WALD	0.28	(0.19–0.58)	0.25	(0.13–0.44)	0.011	(0.008–0.014)	0.298	(0.109–1.444)	207.11	0.56	
	Log-norm	0.25	(0.17–0.43)	0.84	(0.63–1.18)	0.011	(0.008–0.014)	0.298	(0.109–1.442)	207.47	1.26	
	Exp-pow	0.27	(0.18–0.45)	0.56	(0.29–0.96)	0.011	(0.008–0.015)	0.249	(0.096–1.057)	209.86	6.05	
Sierra de los Porrones	**Log-norm**	**0.23**	**(0.13–0.50)**	**1.07**	**(0.84–1.56)**	**0.021**	**(0.013–0.033)**	**0.369**	**(0.118–2.966)**	**110.01**	–	**0.35**
	2Dt	0.28	(0.16 - *na*)	1.75	(1.27–3.12)	0.021	(0.013–0.033)	0.371	(0.117–3.291)	110.02	0.01	
	Exp-pow	0.21	(0.13–0.40)	0.48	(0.19–0.93)	0.020	(0.013–0.033)	0.370	(0.118–3.227)	110.15	0.27	
	WALD	0.20	(0.10 - *na*)	0.07	(0.03–0.12)	0.024	(0.015–0.043)	0.280	(0.098–1.231)	111.27	2.52	

For each parameter, 95% confidence intervals are presented along with the mean value. Log-norm, log-normal; Exp-pow, exponential-power. *na* denotes that the confidence interval limit is not available because of flat likelihood function. *– logL*, log-likelihood; AIC, Akaike’s information criterion; *r*, Pearson correlation coefficient between observed and predicted seedling density values. Models with the lowest AIC are marked in bold for each population.

Differences in AIC values indicated that no single kernel function provided the best fit across all populations of either of the two species (Table 2 and 3). For *S. ciliata*, the WALD kernel was among the most parsimonious models (i.e. models whose AIC values differ ΔAIC <2 from the model with the lowest AIC [55]) in all five populations, while the log-normal model was among the most parsimonious in four populations, the exponential-power model in three populations and the 2Dt model in two populations (Table 2). Results did not support a particular kernel shape in *S. ciliata*: the mesokurtic, thin-tailed exponential-power with *u* >1 showed similarly good fits (ΔAIC <2) as leptokurtic functions with either exponential (WALD) or fat (2Dt and log-normal) tails within the same populations. Using the WALD kernel across all *S. ciliata* populations, species-level estimates of mean dispersal distance and the shape parameters were *δ = *0.39 m (95% CI: 0.35–0.45 m) and *u* = 0.80 (0.63–1.13). For *A. caespitosa,* the log-normal kernel ranked among the best models in all four populations where estimation converged, whereas the WALD, 2Dt and exponential-power models showed a similarly good performance in three populations (Table 3). Supported models in *A. caespitosa* always included leptokurtic functions with exponential or fat tails (including the exponential-power with *u*<1). Using the log-normal model across all *A. caespitosa* populations, the species-level estimates of kernel parameters were *δ = *0.43 m (0.32–0.60 m) and *u* = 0.98 (0.87–1.14).

The fits of the most parsimonious models ranged from good in *A. caespitosa* populations (*r* ^2^ = 0.17–0.35) to fair or poor (*r* ^2^ = 0.01–0.12) in *S. ciliata* populations (Tables 2 and 3) and were highly significant (*p*<0.0001) in all cases except in the population with the lowest seedling number (*p = *0.32 for the Laguna population of *S. ciliata*, which only had 6 seedlings). Population estimates of mean dispersal distance (*δ*) and fecundity (*β*) parameters were generally consistent when assuming different kernel families (Table 2 and 3). No significant correlation was found between log *β* and log *δ* (*r = *0.35, *p = *0.65).

Focusing on the cross-population comparisons of the WALD dispersal model for *S. ciliata*, estimated mean dispersal distances ranged between *δ* = 0.23 and 0.71 m (Table 2), although differences were only marginally significant (*χ* ^2^_4*d.f.*_ *= *8.846, *p* = 0.065). Estimated shape parameters ranged between *u* = 0.55 and 2.26 and were not significantly different from each other (*χ* ^2^_4*d.f.*_ *= *7.022, *p* = 0.135). Fecundity parameters ranged between *β* = 0.001 and 0.009 seedlings/cm across *S. ciliata* populations and were significantly different (*χ* ^2^_4*d.f.*_ = 254.26, *p*<0.001). Per-population tests indicated that all population estimates of *β* were significantly different from the species average (Table 4).

**Table 4:** Comparison of estimated fecundity parameters (*β*) among *S. ciliata* populations.

Population	*β_i_* (seedlings/cm)	*β-_i_* (seedlings/cm)	−log*L*	*χ* ^2^_1*d.f.*_ (*p-value)*
Peñalara	0.009	0.003	−3276.28	229.28 (<0.0001)*
Cabezas de Hierro	0.003	0.007	−3365.25	51.35 (<0.0001)*
Nevero	0.001	0.006	−3361.72	58.41 (<0.0001)*
Najarra	0.002	0.006	−3366.10	49.64 (<0.0001)*
Laguna	0.001	0.005	−3380.92	19.99 (<0.0001)*
Average	0.005	–	−3390.92	–

*β_i_* is the fecundity parameter for the corresponding population and *β-_i_* is the average *β* across the remaining four populations. –log*L* is the log-likelihood of the model considering *β_i_* and separately *β-_i_*. Last column shows the deviance (*χ* ^2^_1*d.f.*_ -distributed) of the model considering *β_i_* and separately *β-_i_* relative to the model (shown in the last row) assuming constant *β* across all five populations. Asterisks denote a significant test after sequential Bonferroni correction.

Assuming a log-normal dispersal model for *A. caespitosa*, population estimates of *δ* varied between 0.23 and 0.78 m (Table 3) and were significantly different (*χ* ^2^_3*d.f.*_ *= *14.022, *p* = 0.003). Per-population tests showed that only the population with the largest *δ* (Loma de Cabezas with 0.78 m) differed significantly from the species average (Table 5). Estimated shape parameters ranged between *u* = 0.84 and 1.07 and were not significantly different from each other (*χ* ^2^_3*d.f.*_ *= *1.426, *p* = 0.699). Fecundity parameter estimates exhibited significant differences in *A. caespiotsa* (*χ* ^2^_3*d.f.*_ *= *22.51, *p*<0.001), ranging from *β* = 0.011 to 0.024 seedlings/cm. Per-population tests indicated that maximum estimates of *β* (at Cabezas de Hierro) and minimum estimates of *β* (at Collado de las Vacas) were significantly higher and lower than the species average, respectively (Table 6).

**Table 5:** Comparison of estimated mean dispersal distances (*δ*) among *A. caespitosa* populations.

Population	*δ_i_* (m)	*δ-_i_* (m)	-log*L*	*χ* ^2^_1*d.f.*_ (*p-value)*
Cabezas de Hierro	0.33	0.49	1155.80	1.84 (0.1744)
Loma de Cabezas	0.78	0.29	1150.46	12.53 (0.0003)*
Collado de las Vacas	0.25	0.50	1154.47	4.50 (0.0338)
Sierra de los Porrones	0.23	0.47	1155.20	3.04 (0.0809)
Average	0.43	–	1156.72	–

*δ_i_* is the mean dispersal distance for the corresponding population and *δ-_i_* is the average *δ* across the three remaining populations. –log*L* is the log-likelihood of the model considering *δ_i_* and separately *δ-_i_*. The last column shows the deviance (*χ* ^2^_1*d.f.*_ -distributed) of the model considering *δ_i_* and separately *δ-_i_* relative to the model (shown in the last row) assuming constant *δ* across all four populations. The asterisk denotes a significant test after sequential Bonferroni correction.

**Table 6:** Comparison of estimated fecundity parameters (*β*) among *A. caespitosa* populations.

Population	*β_i_* (seedlings/cm)	*β-_i_* (seedlings/cm)	-log*L*	*χ* ^2^_1*d.f.*_ (*p-value)*
Cabezas de Hierro	0.024	0.013	1152.64	16.65 (<0.0001)*
Loma de Cabezas	0.014	0.020	1159.56	2.82 (0.0932)
Collado de las Vacas	0.011	0.021	1153.55	14.84 (0.0001)*
Sierra de los Porrones	0.021	0.017	1160.66	0.61 (0.4337)
Average	0.017	–	1160.97	–

*β_i_* is the fecundity parameter for the corresponding population and *β-_i_* is the average *β* across the three remaining populations. –log*L* is the log-likelihood of the model considering *β_i_* and separately *β-_i_*. The last column shows the deviance (*χ* ^2^_1*d.f.*_ -distributed) of the model considering *β_i_* and separately *β-_i_* relative to the model (shown in the last row) assuming constant *β* across all four populations. Asterisks denote significant tests after sequential Bonferroni correction.

## Discussion

We used an inverse modelling approach to analyse the variation in effective seed dispersal patterns of two coexisting species along an altitudinal gradient in a high mountain pasture community of central Spain. Estimated mean dispersal distances were short in both species, as most seedlings established less than a meter from the source. No single kernel function provided the best fit across all populations. Furthermore, fecundity parameters were significantly different among populations in both species. Mean dispersal distance was significantly different among *A. caespitosa* populations, while differences in mean dispersal distance among *S. ciliata* populations were only marginally significant. The kernel shape parameter did not vary significantly in either of the two species.

The inverse modelling approach proved to be useful in characterizing effective seed dispersal patterns in both species. Parameter estimates were consistent among populations and species independent of the assumed kernel. In addition, estimates conformed to independent fecundity measures for the same species [30], [34], and to primary seed dispersal estimates obtained with direct and indirect methods in congeneric species (*Silene latifolia*: 0.17–0.85 m, [63]; *Armeria maritima*: 0.6 m, [64]). Although *r* ^2^ values were slightly lower than those obtained in analogous studies [13], [18], [20], they were highly significant (except in the population with the lowest seedling number). This suggests that inverse modelling was able to provide information on the scale of dispersal in the studied high mountain plants, even though there seemed to be substantial distance-independent fluctuations in dispersal and establishment probabilities that cannot be characterized by simple kernel fits as frequently found in heterogeneous environments [19].

Most effectively dispersed seeds were established near conspecific adult plants (Figure S1). Consequently, estimated mean dispersal distances were low, suggesting that both *A. caespitosa* and *S. ciliata* have low effective seed dispersal ranges. Effective dispersal scale is influenced by factors operating at the level of both primary seed dispersal and germination and establishment. According to mechanistic wind dispersal models based on the WALD kernel, the seeds of six high-mountain species are transported no farther than a few meters by primary wind dispersal, probably due to low seed release height [65]. Release height, determined by inflorescence length, is indeed low (about 5–25 cm) in both *A. caespitosa* and *S. ciliata* (see Castroviejo *et al.* [66] for morphological characteristics). However, according to the mechanistic simulations in Dullinger *et al.* [65], the primary seed dispersal range of these two species should be longer than that estimated by our effective dispersal models. This suggests that effective seed dispersal is further limited by post-dispersal processes. Environmental harshness in summer might be the main factor in reducing primary seed dispersal distance. In the two species studied here, recruitment is mainly limited at the seedling emergence and survival stages by summer drought [30], [34]. Furthermore, García-Camacho *et al.* [30] found a positive effect of *A. caespitosa* adult cover on seedling emergence and survival in 0.5×0.5 m samplings subplots. This suggests that adults act as nurse plants, facilitating seedling emergence and survival along the altitudinal gradient [30], as observed in other Mediterranean mountain regions [67]. The effect of adult cover on post-dispersal survival might lead to unrealistic conclusions of short effective dispersal distance, because our modelling approach assumes a distance-invariant survival function. This assumption may be violated in the case of distance-dependent survival because the primary and effective seed dispersal kernel would attain different shapes [68]. Hence, the modelling approach will implicitly assume that seedlings close to a conspecific adult originate from this adult, even though facilitation can also favour seeds from other adults after more substantial dispersal [68]. Given the mean distance between the adult plants recorded in our study plots (mean ± SD: 5.35±0.38 m for *S. ciliata;* 5.21±0.58 m for *A. caespitosa*) and the essentially barochorous seed dispersal mechanism, the ratio of seeds from other adults to seeds from the closest adult is likely to be very small, and, therefore, the effect of this bias negligible. Moreover, all the studied *S. ciliata* populations exhibited significant positive fine-scale spatial genetic structure (FSGS) at the 0–1 m distance class (Lara-Romero *et al.* unpublished data), indicating that the spatial aggregation of genotypes is consistent with the low effective dispersal distance observed in our study.

Mean dispersal distance provides a limited characterization of dispersal range [19], [69]. Other characteristics of the dispersal kernel such as the kurtosis and fatness of the distribution tail may have important demographic, ecological and genetic consequences [12], [19], [69], [70]. Our results did not support a particular kernel shape in the case of *S. ciliata*. Although the behavior near the origin and the tail is not independent in the assumed phenomenological kernels (see [16] and references therein), the virtual absence of seedlings beyond the close proximity of *S. ciliata* adults (Figure S1) may result in similarly good fits for kernels with very different tails but with sufficiently fast probability decay near the origin. That is, fat-tailed kernels could fit well in *S. ciliata*, because the actual dispersal probability decreased fast near the origin and not necessarily because the actual process had a fat tail. In *A. caespitosa*, results supported leptokurtic functions with exponential or fat tails with consistently more abundant isolated seedlings than for *S. ciliata* (Figure S1). This would suggest a larger proportion of long-distance effective dispersal events in *A. caespitosa* than in *S. ciliata*.

The few studies that have previously used inverse modelling with a multi-population approach found local variation in dispersal parameter estimates within species [5], [20]. In Clark *et al.* [5] and LePage *et al.* [20], variation in dispersal patterns within species was found across tree stands of temperate forests that spanned gradients in moisture and canopy openness. Similarly, we expected higher effective dispersal ranges in high-altitude populations because less stressful conditions could reduce the intensity of the adult nurse effect on the survival of emerged seedlings. Furthermore, the lower encroachment by subalpine shrubs at high elevation sites [71] would provide a more open habitat with greater exposure to wind that might favour longer-distance seed dispersal. However, contrary to our expectations, differences in the shape parameter value were not significant among populations in either of the two species. Only the *A. caespitosa* population with the largest mean dispersal distance differed significantly from the species average value for this parameter. Despite the strong variation in demographic structure and/or physical environment along the altitudinal gradient, dispersal parameter estimates tended to be consistent among populations within the species. This implies that the inherent seed dispersal traits of the species (e.g., inflorescence length, fruit morphology, seed size and shape), which are essentially the same in all populations, were the most important factors in determining the effective dispersal kernel in our study sites during the study period. Post-dispersal processes controlling seedling emergence and establishment seem to operate similarly across populations, regardless of evident variation in local demographic and environmental conditions (intra and interspecific plant density, local topography, soil moisture, temperature regime, etc.). This provides new insight into the prevalence and magnitude of intraspecific dispersal variation. Although our results are consistent with previous studies [5], [20], showing that effective mean dispersal distances vary among populations of particular species, they also indicate that effective dispersal kernels can remain invariant across populations of other co-occurring species, even if there is significant variation in demographic structure and the environment.

Effective fecundity varied significantly across populations in both species, and per-population tests identified that these differences were due to the greater fecundity values found in the populations located at the highest altitude. As expected, the fecundity parameter was related to the ratio of seedlings to reproductive adults (Kendall rank correlation coefficient: *τ* = 1, *p*<0.001 for *S. ciliata*; and τ = 0.67, *p* = 0.167 for *A. caespitosa*), which represents a measure of the average number of successfully established seedlings produced by each mother. By contrast, the fecundity parameter was not associated with mean seed production per adult plant (as estimated with a seed-crop sample from a small number of individuals of each population; data not shown). Furthermore, seedling density was positively related to altitude (*τ* = 0.8, *p*<0.05 for both species). These results suggest different seedling mortality rates at different altitudes, which have probably influenced the observed differences in the fecundity parameter among populations. These results are also congruent with previous studies on the demography of the two species [30], [33], indicating that more benign conditions at the populations located at the highest altitudes allow the emergence and establishment of larger numbers of seedlings. Hence, the altitudinal gradient appears to influence effective seed dispersal patterns in *S. ciliata* and *A. caespitosa* through variation in effective seedling establishment probabilities, but not as much through variation in the effective seed dispersal range. The explicit incorporation of finer-scale environmental factors and landscape features (e.g. shrub cover) in our models (for instance through the application of the movement space concept; Schurr *et al.* [14]) might help to estimate and model the potential effects of environmental variables on fine-scale spatial patterns of effective dispersal more accurately, and thus increase the amount of explained variance in within-population recruitment patterns.

The estimates provided by empirical dispersal studies such as this one could help to explicitly incorporate real migration constraints in predictive species distribution models. This information is all the more relevant because accurate predictions about dispersal and migration capacities are considered to be among the most significant uncertainties in projecting climate impacts on plant species ranges [72]. Our results, together with previous seed dispersal mechanistic simulations of alpine plant species [65] show that the majority of dispersal events occur within a few meters from the source. This is consistent with [73], who found widespread post-glacial dispersal constraints on the current distribution of plants in the European Alps. These findings raise doubts about the capacity of high mountain plants to track their climatic niche under the rapid climate warming predicted for mountain systems during the 21^st^ century [23]. However, spatial spread and colonization rates are not necessarily governed by mean seed dispersal distance but by the frequency of rare long-distance dispersal (LDD) events [11], [74], [75]. LDD is usually caused by extreme events in terms of horizontal wind speed or turbulence [21], [74]. High-mountain habitats are exposed to frequent and strong updrafts [76], [77]. Consequently, LDD may occur frequently in high-mountain environments [78]. These events could greatly increase the chance of threatened populations to track the altitudinal-zone displacement induced by warming. Therefore, further research should accurately estimate the impact of LDD on seed dispersal patterns in mountain ecosystems using adequate methods (for instance through the application of recently developed genetic methods [79]–[81]).

### Conclusions

This study shows that there is not a single kernel function that consistently provides the best fits across species and populations. More importantly, this study points out that effective dispersal kernels can remain invariant across populations of particular species under strong variation in demographic structure and/or physical environment, while they may vary among populations of other co-occurring species. These results call for a case-by-case analysis in a wider range of taxa and environments to assess the validity of approaches that assume invariant species-specific dispersal kernels [22].

## Supporting Information

Figure S1 Spatial distribution of adults (circles) and seedlings (crosses) in each study plot of *A. caespitosa* and *S. ciliata*.
(PDF)

Table S1 Estimated parameters for the models fitted to the seedling recruitment data of *Silene ciliata* (cell size length 0.25 and 0.50 m). *δ*, mean dispersal distance (m); *u* shape parameter; *β*, fecundity parameter (seedlings/cm); *θ*, negative binomial parameter; – logL, log-likelihood; *nc* denotes models that did not converge.
(PDF)

Table S2 Estimated parameters for the four models fitted to the seedling recruitment data of *Armeria caespitosa*. (cell size length 0.25 and 0.50 m). *δ*, mean dispersal distance (m); *; u* shape parameter; *β*, fecundity parameter (seedlings/cm); *θ*, negative binomial parameter; – logL, log-likelihood; *nc* denotes models that did not converge.
(PDF)
