# Supplementary material for: Assessing Intraspecific Variation in Effective Dispersal Along an Altitudinal Gradient: A Test in Two Mediterranean High-Mountain Plants
Source: PLoS One. 2014 Jan 29;9(1):e87189. doi: 10.1371/journal.pone.0087189 (PMC3906119; doi:10.1371/journal.pone.0087189)
Supplement: Figure S1 — Spatial distribution of adults (circles) and seedlings (crosses) in each study plot of A. caespitosa and S. ciliata. (PDF) [file pone.0087189.s001.pdf]

Figure S1. Spatial distribution of adults (circles) and seedlings (crosses) in each study plot of *A. caespitosa* and *S. ciliata*.

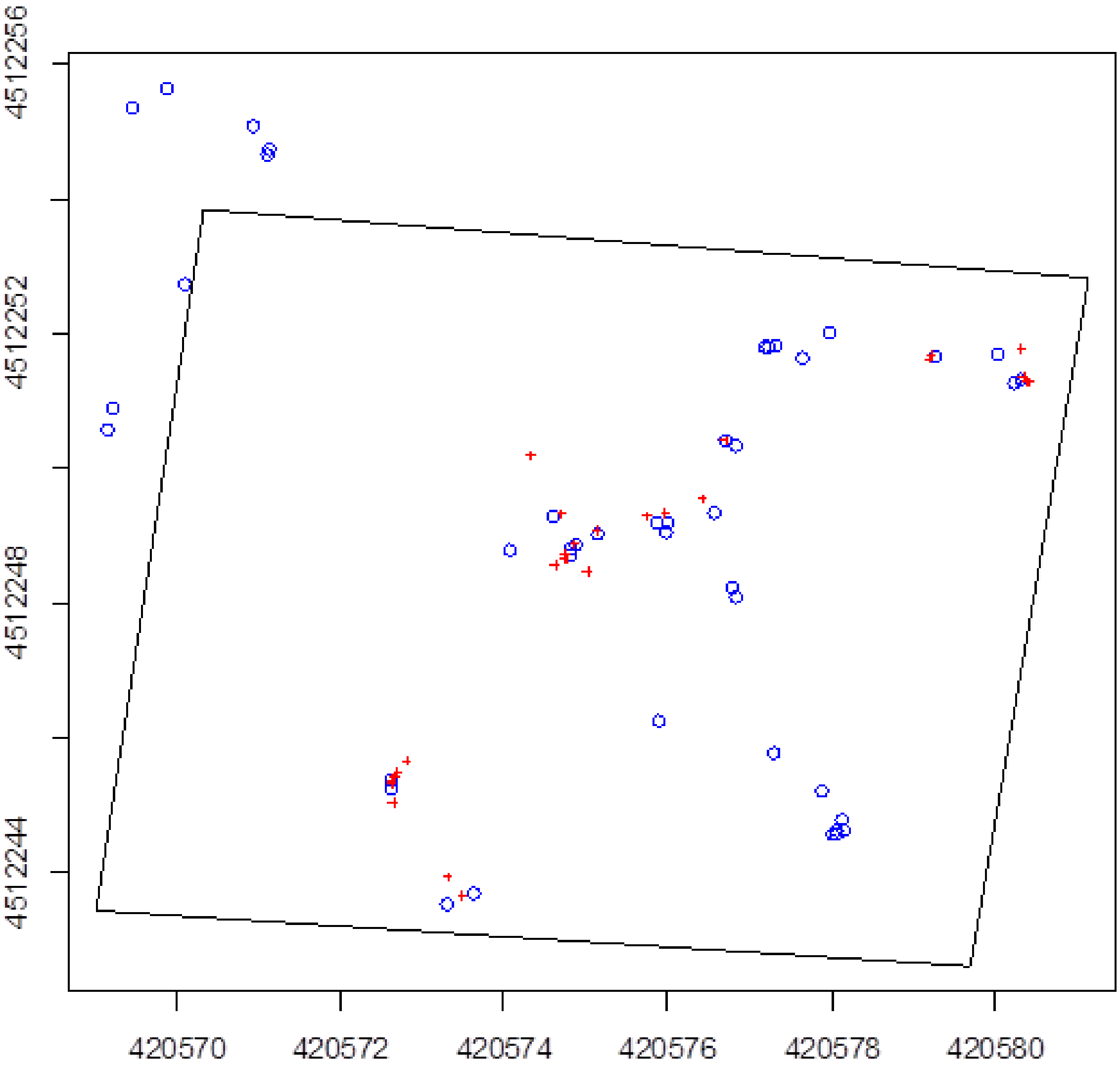

Figure S1. (A) Spatial distribution of adults (circles) and seedlings (crosses) in the Sierra de los Porrones population of *Armeria caespitosa*. Axes show UTM coordinates in meters.

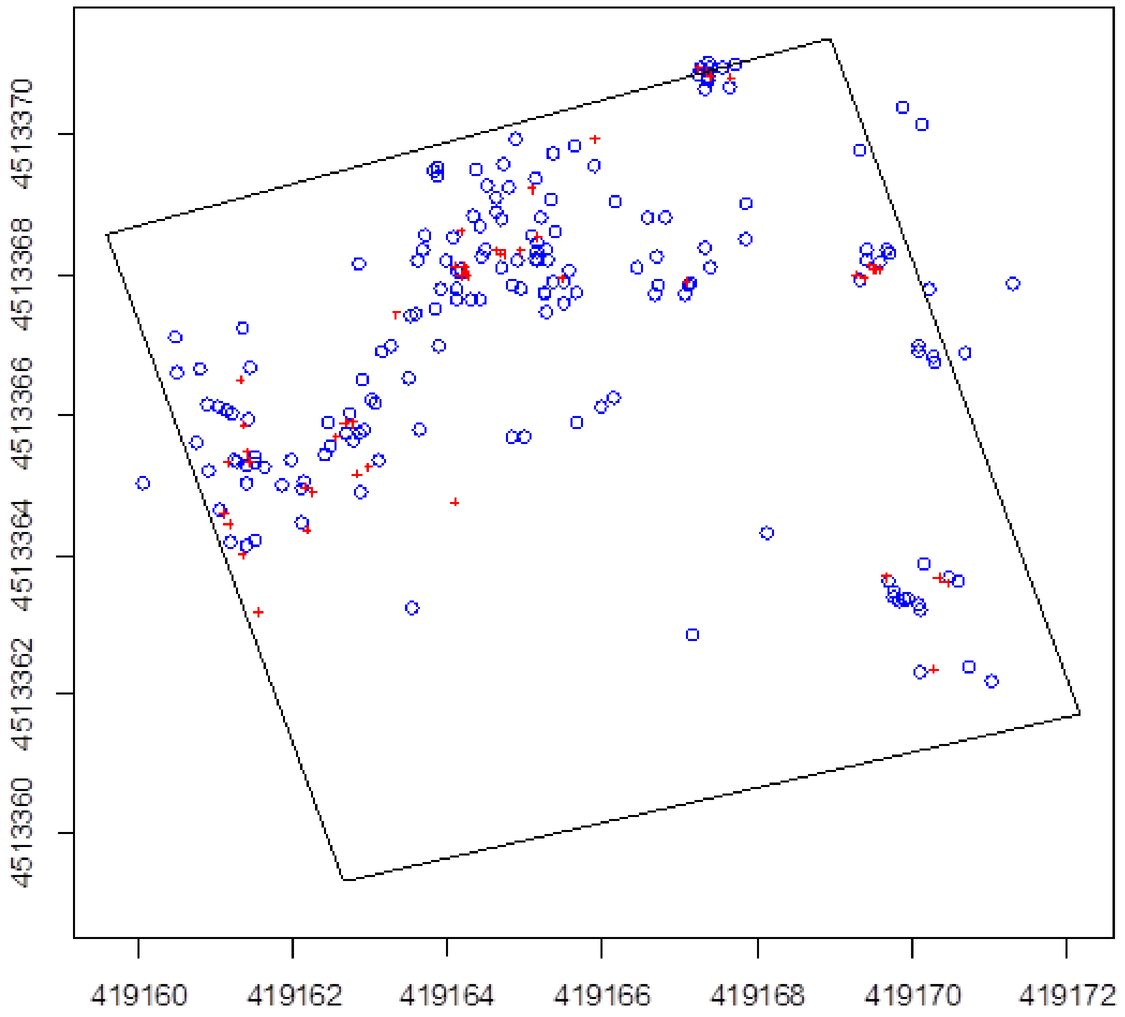

Figure S1. (B) Spatial distribution of adults (circles) and seedlings (crosses) in the Collado de las Vacas population of *Armeria caespitosa*. Axes show UTM coordinates in meters.

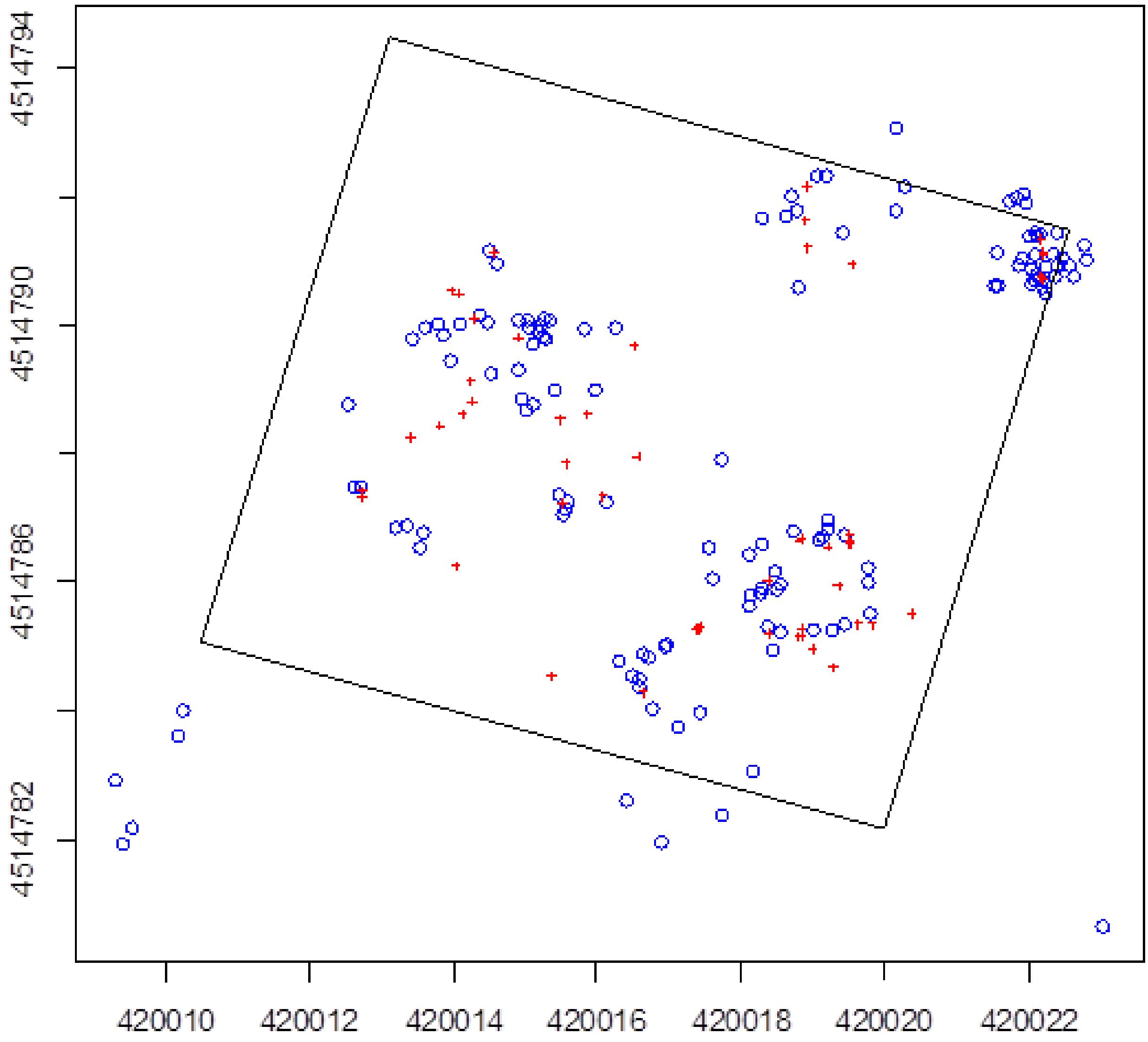

Figure S1. (C) Spatial distribution of adults (circles) and seedlings (crosses) in the Loma de Cabezas population of *Armeria caespitosa*. Axes show UTM coordinates in meters.

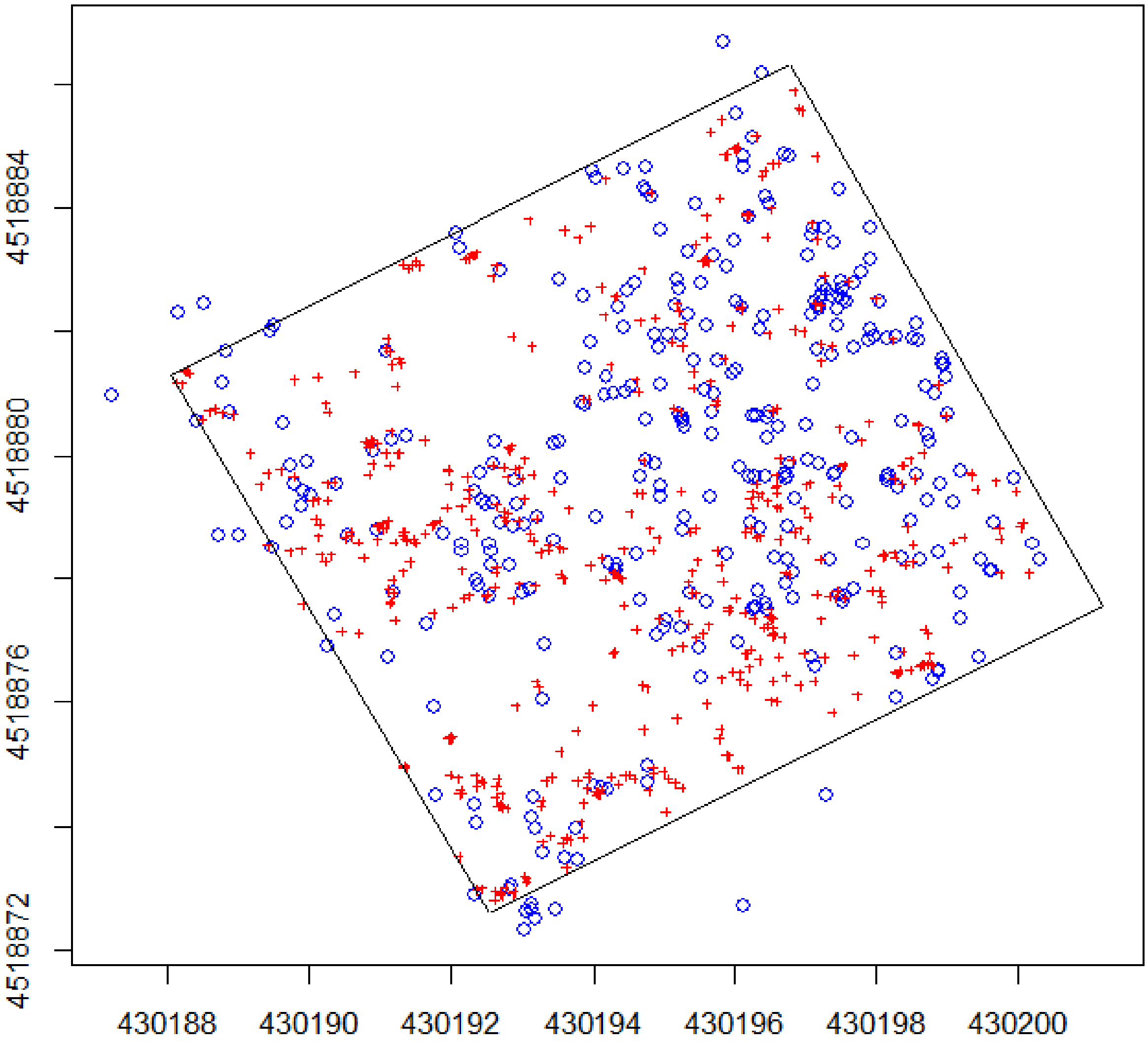

Figure S1. (D) Spatial distribution of adults (circles) and seedlings (crosses) in the Najarra population of *Armeria caespitosa*. Axes show UTM coordinates in meters.

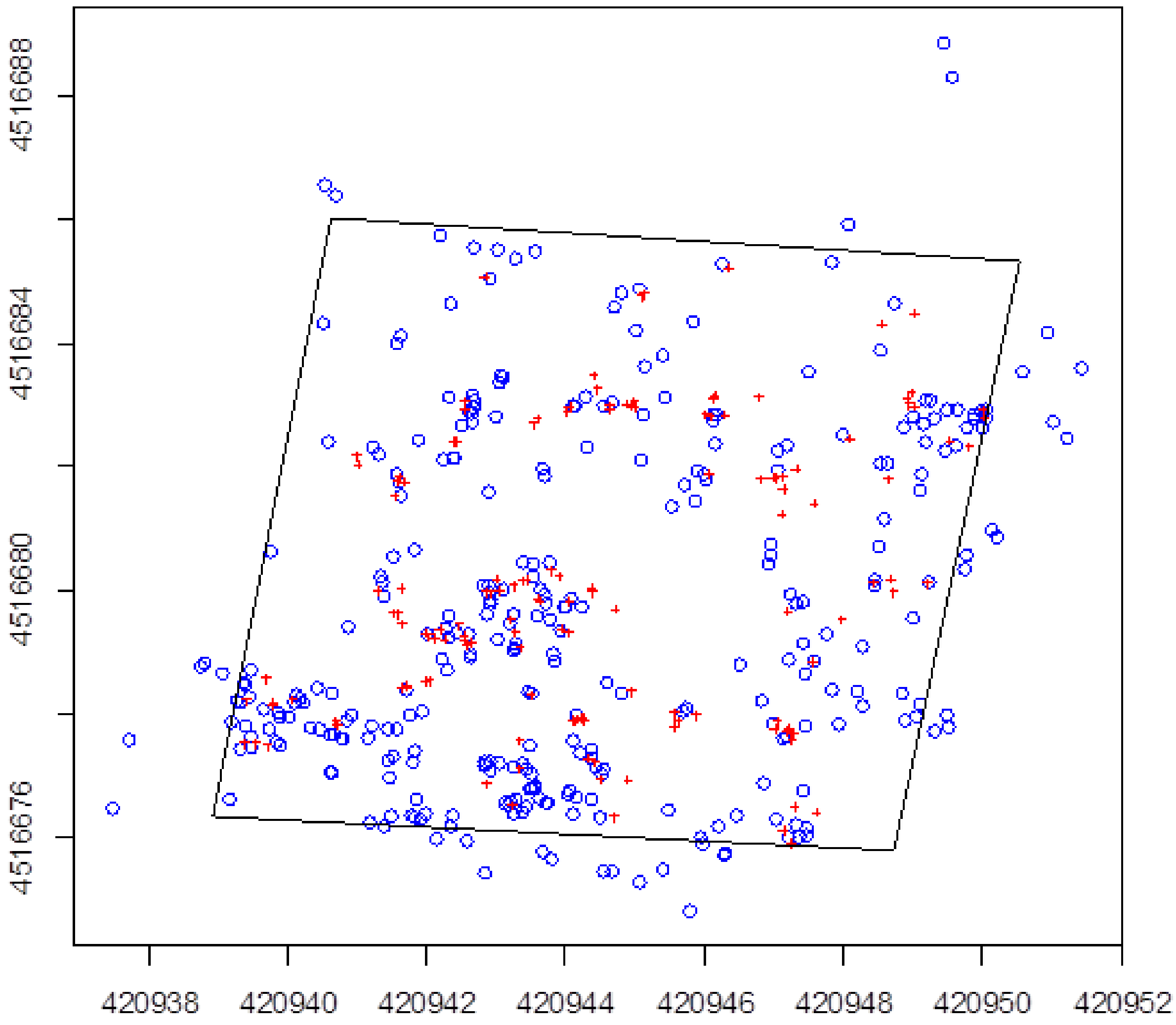

Figure S1. (E) Spatial distribution of adults (circles) and seedlings (crosses) in the Cabezas de Hierro population of *Armeria caespitosa*. Axes show UTM coordinates in meters.

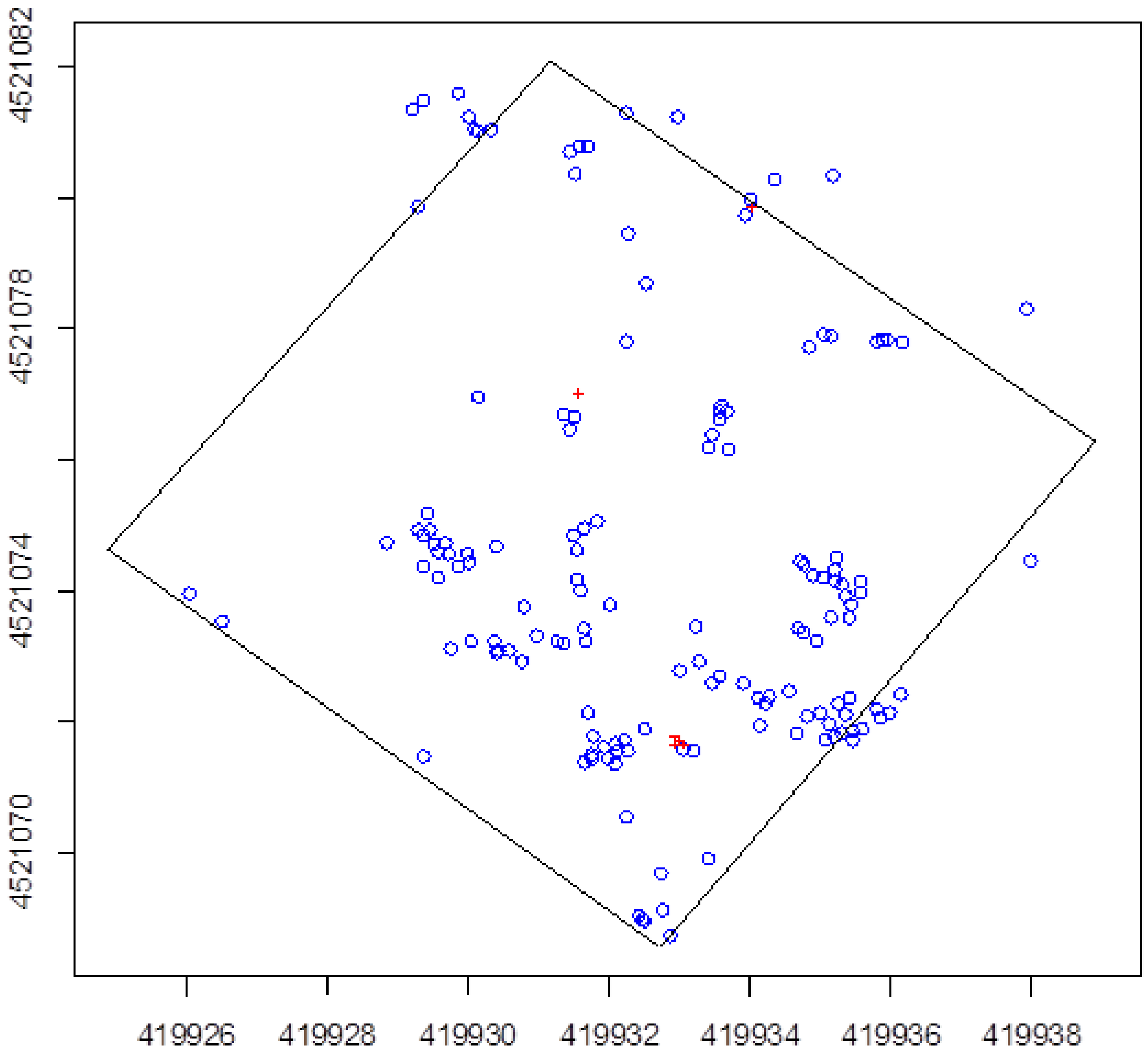

Figure S1. (F) Spatial distribution of adults (circles) and seedlings (crosses) in the Cabezas de Hierro population of *Silene ciliata*. Axes show UTM coordinates in meters.

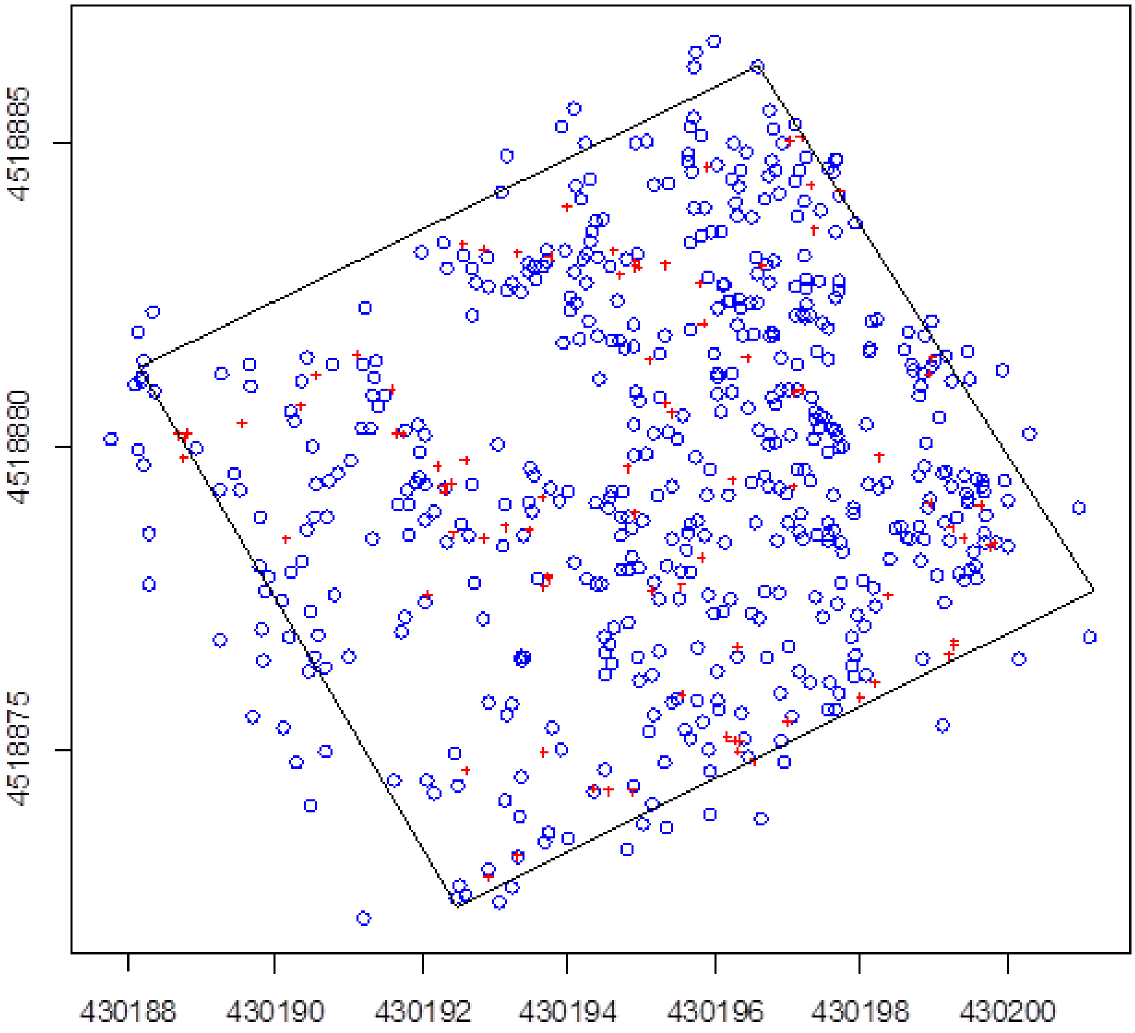

Figure S1. (G) Spatial distribution of adults (circles) and seedlings (crosses) in the Najarra population of *Silene ciliata*. Axes show UTM coordinates in meters.

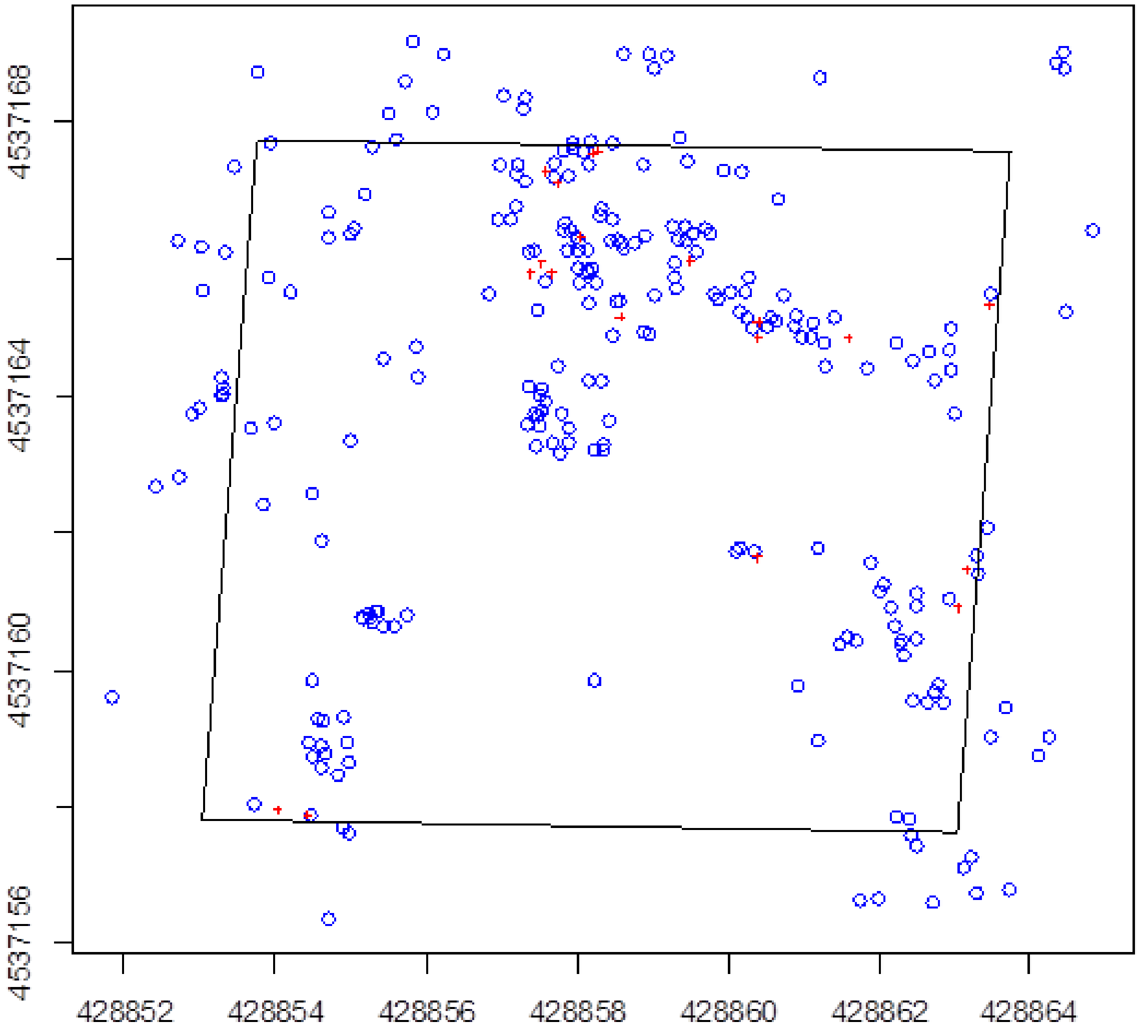

Figure S1. (H) Spatial distribution of adults (circles) and seedlings (crosses) in the Nevero population of *Silene ciliata*. Axes show UTM coordinates in meters.

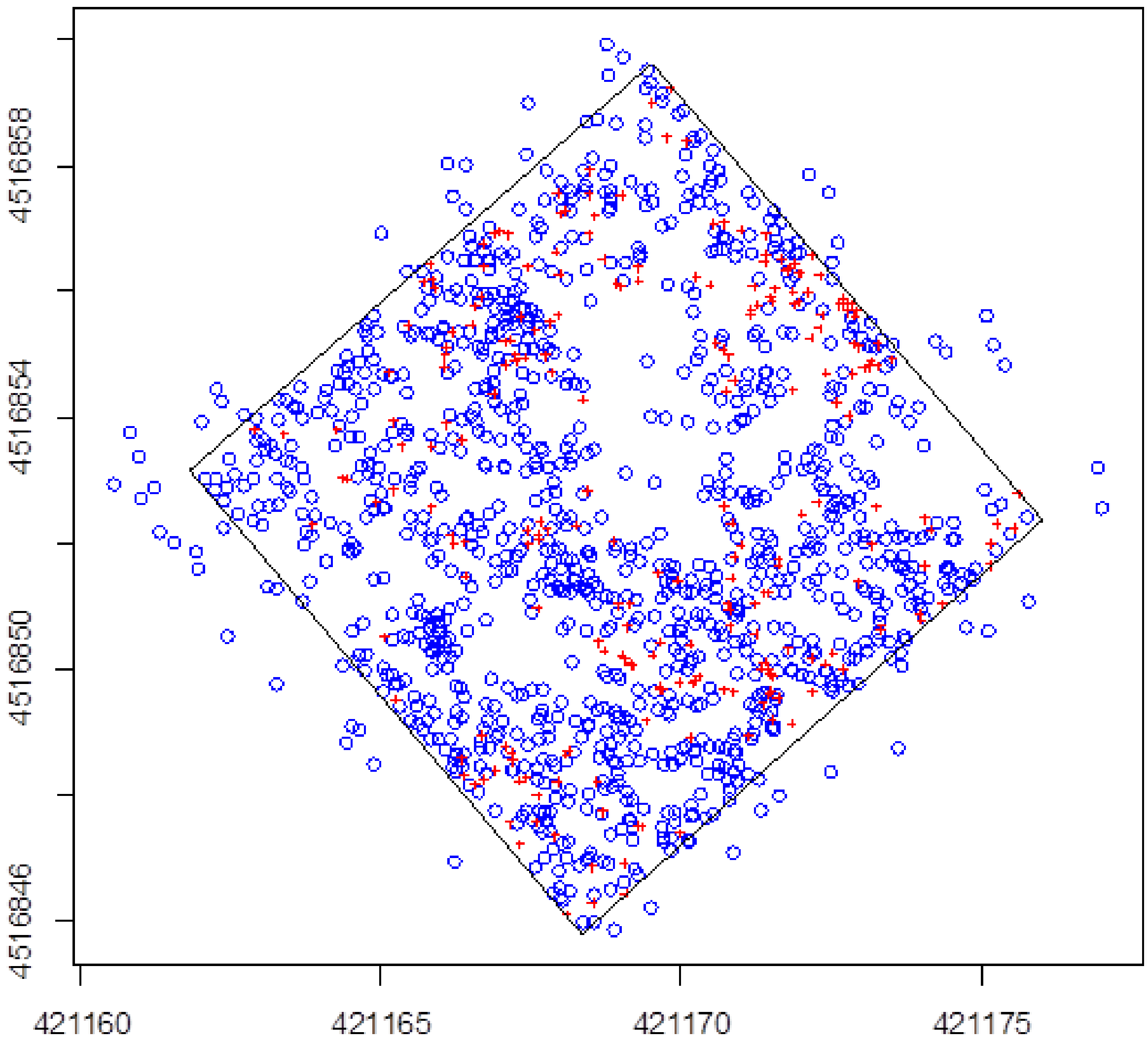

Figure S1. (I) Spatial distribution of adults (circles) and seedlings (crosses) in the Cabezas de Hierro population of *Silene ciliata*. Axes show UTM coordinates in meters.

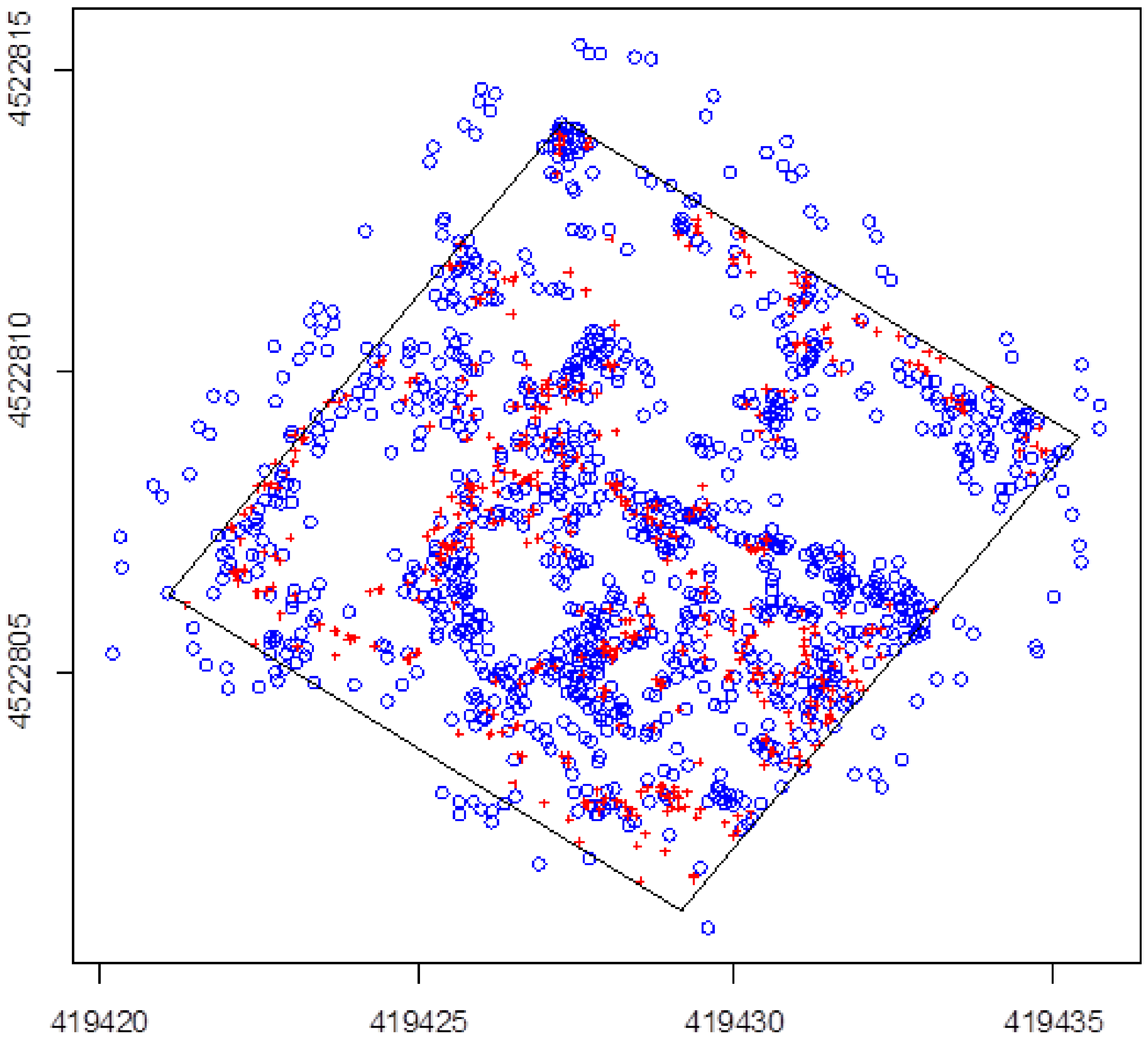

Figure S1. (J) Spatial distribution of adults (circles) and seedlings (crosses) in the Peñalara population of *Silene ciliata*. Axes show UTM coordinates in meters.
