# Supplementary material for: Assessing Intraspecific Variation in Effective Dispersal Along an Altitudinal Gradient: A Test in Two Mediterranean High-Mountain Plants
Source: PLoS One. 2014 Jan 29;9(1):e87189. doi: 10.1371/journal.pone.0087189 (PMC3906119; doi:10.1371/journal.pone.0087189)
Supplement: Table S1 — Estimated parameters for the models fitted to the seedling recruitment data of Silene ciliata (cell size length 0.25 and 0.50 m). δ, mean dispersal distance (m); u shape parameter; β, fecundity parameter (seedlings/cm); θ, negative binomial parameter; – logL, log-likelihood; nc denotes models that did not converge. (PDF) [file pone.0087189.s002.pdf]

Table S1. Estimated parameters for the models fitted to the seedling recruitment data of *Silene ciliata (*cell size length 0.25 and 0.50 m)

		0.25 m					0.50 m				
population	kernel	δ	*u*	β	θ	- log*L*	δ	*u*	β	θ	- log*L*
Peñalara	2Dt	0.33	3.20	0.010	0.511	1034.26	0.34	7.15	0.010	0.87	556.65
	Log-norm	0.34	0.66	0.010	0.511	1035.41	0.36	0.76	0.010	0.87	558.06
	WALD	0.34	0.68	0.010	0.510	1035.79	0.36	0.42	0.010	0.86	559.07
	Exp-pow	0.32	1.21	0.010	0.509	1034.49	0.34	1.54	0.010	0.87	556.60
Cabezas de Hierro	2Dt	0.42	172.46	0.003	0.523	708.02	0.41	5.16	0.003	0.91	420.42
	Log-norm	0.42	0.50	0.003	0.531	706.53	0.43	0.62	0.003	0.91	420.47
	WALD	0.42	1.49	0.003	0.531	706.48	0.43	1.09	0.003	0.91	420.69
	Exp-pow	0.41	2789.58	0.003	0.524	705.73	0.40	1.70	0.003	0.90	420.51
Nevero	2Dt	0.25	10.44	0.001	142.159	82.84	0.44	172.60	0.001	0.72	60.14
	log-norm	0.25	0.49	0.001	142.154	82.12	0.67	0.12	0.001	1.30	58.69
	WALD	0.25	0.89	0.001	142.150	82.07	0.67	47.81	0.001	1.48	58.69
	Exp-pow	0.25	2.10	0.001	142.053	82.85	0.56	398.50	0.001	0.96	59.05
Najarra	2Dt	*nc*	*nc*	*nc*	*nc*	*nc*	*nc*	*nc*	*nc*	*nc*	*Nc*
	Log-norm	*nc*	*nc*	*nc*	*nc*	*nc*	*nc*	*nc*	*nc*	*nc*	*Nc*
	WALD	*nc*	*nc*	*nc*	*nc*	*nc*	*nc*	*nc*	*nc*	*nc*	*Nc*
	Exp-pow	0.72	0.47	0.002	0.230	337.85	0.99	0.33	0.002	0.50	225.73
Laguna	2Dt	0.25	172.44	0.001	0.003	23.23	0.25	1.76	0.001	0.02	19.30
	Log-norm	0.28	0.36	0.001	0.003	23.24	0.24	0.76	0.001	0.02	19.29
	WALD	0.27	1.89	0.001	0.003	23.22	0.24	0.33	0.001	0.02	19.24
	Exp-pow	0.29	5.87	0.001	0.004	23.11	0.20	0.58	0.001	0.02	19.30

δ, mean dispersal distance (m); *u* shape parameter; β, fecundity parameter (seedlings/cm); θ, negative binomial parameter; – logL, log-likelihood; *nc* denotes models that did not converge.
