# Supplementary material for: Assessing Intraspecific Variation in Effective Dispersal Along an Altitudinal Gradient: A Test in Two Mediterranean High-Mountain Plants
Source: PLoS One. 2014 Jan 29;9(1):e87189. doi: 10.1371/journal.pone.0087189 (PMC3906119; doi:10.1371/journal.pone.0087189)
Supplement: Table S2 — Estimated parameters for the four models fitted to the seedling recruitment data of Armeria caespitosa. (cell size length 0.25 and 0.50 m). δ, mean dispersal distance (m); ; u shape parameter; β, fecundity parameter (seedlings/cm); θ, negative binomial parameter; – logL, log-likelihood; nc denotes models that did not converge. (PDF) [file pone.0087189.s003.pdf]

Table S2. Estimated parameters for the four models fitted to the seedling recruitment data of *Armeria caespitosa*. (cell size length 0.25 and 0.50 m).

		0.25 m					0.50 m				
population	kernel	δ	u	β	θ	- log*L*	δ	u	β	θ	- log*L*
Cabezas de Hierro	2Dt	0.40	1.91	0.024	0.261	415.97	0.40	7.20	0.024	0.80	268.95
	Log-norm	0.34	0.84	0.024	0.259	415.96	0.43	0.61	0.024	0.80	268.62
	WALD	0.34	0.40	0.024	0.256	416.10	0.43	0.96	0.024	0.80	268.74
	Exp-pow	0.31	0.78	0.024	0.257	416.41	0.39	1.85	0.024	0.80	269.01
Najarra	2Dt	*nc*	*nc*	*nc*	*nc*	*nc*	*nc*	*nc*	*nc*	*nc*	*nc*
	Log-norm	*nc*	*nc*	*nc*	*nc*	*nc*	*nc*	*nc*	*nc*	*nc*	*nc*
	WALD	*nc*	*nc*	*nc*	*nc*	*nc*	*nc*	*nc*	*nc*	*nc*	*nc*
	Exp-pow	*nc*	*nc*	*nc*	*nc*	*nc*	*nc*	*nc*	*nc*	*nc*	*nc*
Loma Cabezas	2Dt	0.65	2.88	0.014	0.196	185.10	0.69	2.28	0.014	0.65	123.64
	Log-norm	0.82	1.14	0.014	0.225	184.52	0.92	1.21	0.014	0.76	123.30
	WALD	0.73	0.40	0.014	0.219	184.84	0.67	1.26	0.014	0.60	124.43
	Exp-pow	0.65	0.58	0.014	0.224	183.92	0.67	0.52	0.014	0.77	122.65
Collado de las Vacas	2Dt	0.41	1.75	0.011	0.463	151.63	0.43	1.85	0.010	0.75	99.30
	Log-norm	0.32	0.98	0.011	0.458	152.05	0.34	0.77	0.010	0.84	99.19
	WALD	0.33	0.18	0.011	0.424	152.79	0.36	0.41	0.010	0.85	98.63
	Exp-pow	0.31	0.54	0.011	0.455	152.64	0.37	0.66	0.010	0.68	100.90
Sierra de los Porrones	2Dt	0.25	1.84	0.022	0.387	75.93	0.26	3.58	0.024	0.46	54.49
	Log-norm	0.22	0.88	0.021	0.396	76.01	0.28	0.50	0.023	0.57	53.51
	WALD	0.23	0.20	0.021	0.417	75.79	0.28	0.99	0.023	0.57	53.59
	Exp-pow	0.20	0.43	0.024	0.350	76.60	0.27	1.32	0.023	0.45	55.04

δ, mean dispersal distance (m); u shape parameter; β, fecundity parameter (seedlings/cm); θ, negative binomial parameter; – logL, log-likelihood; *nc* denotes models that did not converge.
